# The HIV epidemic in Colombia: spatial and temporal trends analysis

**DOI:** 10.1186/s12889-021-10196-y

**Published:** 2021-01-21

**Authors:** Jhon Freddy Montana, Glenda Roberta Oliveira Naiff Ferreira, Carlos Leonardo Figueiredo Cunha, Ana Angélica Rêgo de Queiroz, Wellington Augusto Andrade Fernandes, Sandra Helena Isse Polaro, Lucia Hisako Takase Gonçalves, Danielle Costa Carrara Couto, Elucir Gir, Renata Karina Reis, Wiliam Sorensen, Eliã Pinheiro Botelho

**Affiliations:** 1 Nursing Graduate Program, Federal University of Para, Rua Augusto Correia, 01, Complexo Saúde, Guamá, Belém, Para 66075-110 Brazil; 2 Nursing Department of Federal University of Rio Grande do Norte, Centro das Ciências da Saúde, Campus Universitário Lagoa Nova, Natal, Rio Grande do Norte 59078-970 Brazil; 3 Laboratory of Spatial Analyzes (LAENA), Center for Amazonina Studies (NAEA), Federal University of Para, Rua Ausgusto Correia, 01, Complexo Engenharia, Guamá, Belém, Para 66075-110 Brazil; 4 School of Technology in Geoprocessing, Federal University of Pará, Rua Augusto Correia, 01, Complexo Engenharia, Guamá, Belém, Para 66075-110 Brazil; 5 Graduate Program of Fundamental Nursing. Nursing School of Ribeirao Preto, University of Sao Paulo, Av dos Bandeirantes, 3900. Campus Universitario – Monte Alegre, Ribeirao Preto, Sao Paulo, 14040-902 Brazil; 6 Department of Health & Kinesiology, University of Texas at Tyler, 3900 University Blvd., Tyler, TX 75799 USA

**Keywords:** HIV, Acquired immunodeficiency syndrome, Colombia, South America, Spatial analysis

## Abstract

**Background:**

Colombia has the fourth highest incidence rate of HIV/AIDS among all Latin American countries and it has been increasing since the 1980s. However, the number of studies that addresses this trend is limited. Here, we employed spatial and temporal trend analyses to study the behaviour of the epidemic in the Colombian territory.

**Methods:**

Our sample included 72,994 cases of HIV/AIDS and 21,898 AIDS-related deaths reported to the National Ministry of Health between 2008 and 2016. We employed the joinpoint regression model to analyse the annual HIV/AIDS incidence and AIDS mortality rates. In the spatial analysis, we used univariate autocorrelation techniques and the Kernel density estimator.

**Results:**

While the HIV/AIDS incidence had an increasing trend in Colombia, the AIDS mortality rate was stable. HIV/AIDS incidence and AIDS mortality showed a downward trend in the 0–14 age group. An upward trend was observed for HIV/AIDS incidence in people older than 15 years and with the highest trend in the 65 years and above group. AIDS mortality showed an increasing trend among people aged 65 years or older. The comparison between the sexes showed an upward trend of HIV/AIDS incidence in all age groups and AIDS-mortality rates in 65 years and above in men, while in women, the incidence was upward among those aged 45 years and above, and concerning the AIDS-mortality rate in the 45–64 group. The high–high clusters of HIV/AIDS incidence and AIDS mortality were located in the Andean and Caribbean regions.

**Conclusion:**

Our study found an upward trend in HIV/AIDS incidence and a stable trend in the AIDS mortality rate in Colombia. The downward trend in HIV/AIDS incidence and AIDS mortality rate in the 0–14 age group reflects the downwards mother-to-child HIV transmission. The upward trend in HIV/AIDS incidence in older women and AIDS mortality in younger women rates, compared with men, may be due to late diagnosis and treatment. The Caribbean and the ‘coffee belt’ regions were the most impacted by the HIV epidemic, most likely due to sexual tourism. Our results provide crucial information that may help Colombian health authorities fight HIV transmission.

**Supplementary Information:**

The online version contains supplementary material available at 10.1186/s12889-021-10196-y.

## Background

Since 1980, 74.9 million people have been infected by the Human Immunodeficiency Virus (HIV), and 32 million have died by Acquired Immunodeficiency Syndrome (AIDS) [1]. After decades of HIV/AIDS experience, ending HIV transmission remains a challenge to world health authorities. While global AIDS-related deaths have decreased by more than 50% after Antiretroviral Therapy (ART) was implemented, the HIV/AIDS incidence rate achieved only a slight decrease [1–3].

The scarcity of public policies for combatting HIV and economic regional disparities are directly correlated with the increase in the incidence of HIV in Latin America (LA), where 1.9 million people live with HIV and AIDS (PLWHA). Between 2010 and 2018, a 7% increase in new HIV infections was recorded throughout LA [1, 2, 4].

Among all LA countries, Colombia has the fourth highest HIV/AIDS prevalence rate. Its national HIV/AIDS prevalence is approximately 0.7%, but much higher among key populations – men who have sex with men, sex workers, injection drug users and prisoners. The Colombian Caribbean region has the second-highest prevalence of HIV/AIDS in the world after sub-Saharan Africa. By the end of 2019, there were 330,000 Colombian PLWHA, 6900 AIDS-related deaths and 13,000 new HIV infections recorded in the same year. Considering the progress towards 90–90–90 targets proposed by UNAIDS to end HIV by 2030, Colombia reached only 62% of that goal. For example, approximately 50% of Colombian people living with HIV still have not been diagnosed for the virus, and only 55% of PLWHA are on ART. Of those receiving treatment, only 68% are virally suppressed. All Colombians and legal immigrants have access to HIV tests and ART [5].

Several factors contribute to the expansion of HIV in Colombia; such factors include social inequalities with the majority of its population living in poverty and with low access to the healthcare system, social discrimination and stigmatization of PLWHA, the forced displacement of the population by internal armed conflicts, the high level of violence against women and sexual tourism [6–8].

The recent immigration flux of Venezuelans also contributes to the expansion of the HIV epidemic in Colombia by placing a substantial burden on the health care resources of Colombian border municipalities. Given the collapse of the healthcare system in Venezuela, Venezuelans living with HIV migrate to Colombia in search of treatment. Until 2018, approximately 2000 Venezuelans in Colombia were living with HIV. Although immigrants with Colombian Permits of Permanence have full access to the healthcare system, including free ART, the massive influx of Venezuelans into Colombia has led to an overload in administrative processing; many Venezuelan immigrants remain illegal [9, 10]. This phenomenon has impacted Colombia’s healthcare economy. Colombia spends approximately 0.5% of it’s healthcare resources and 1% of it’s social health insurance budget, in HIV treatment alone [11].

The HIV epidemic can be addressed by describing its different dynamics in each Colombian territory given that the epidemic is influenced by sociopolitical and economic factors [2, 12]. Spatial analysis studies are ideal in identifying areas that are highly affected by the epidemic and in highlighting contributing territorial factors. Likewise, temporal trend studies optimize and reveal the impact of public policies against the epidemic [13].

Therefore, this study aimed to analyse the HIV/AIDS epidemic in Colombia by employing spatial and temporal trend analysis techniques. Here, we considered the incidence of HIV/AIDS and AIDS mortality rates.

## Methods

### Study design and settings

This ecological study used secondary data provided by the Colombian Ministry of Health.

**Fig. 1:**
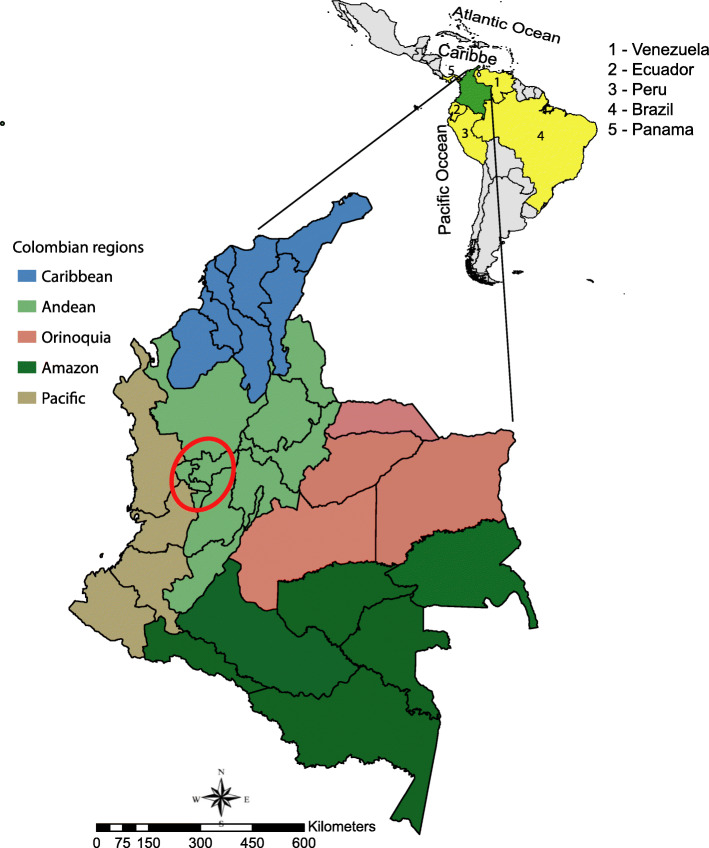
Colombian map showing the provinces limited by regions. The red circle delimits the “Coffee belt” region. The maps were constructed using ArcGis version 10.6.1

Colombia has an estimated population of 50,375,594 in a 2,070,408 km^2^ area. It is divided into five main political regions (the Caribbean, Pacific, Andean, Orinoquia and Amazon regions), 32 provinces and 1123 municipalities. Colombia borders Brazil and Venezuela in the east, Ecuador and Peru in the south, Panama in the northwest and the Caribbean Sea and the Pacific Ocean in the north and west borders, respectively (Fig. 1). Among the LA countries, Colombia has the fourth highest income distribution inequality (GINI index = 0.50). The majority of people live in poverty without secure jobs, having low educational attainment and poor access to healthcare services. Colombia has one of the highest sexual tourism rates compared to other countries worldwide [14], with the Caribbean and ‘coffee belt’ regions being the main tourist destinations. The ‘coffee belt’ comprises the states of Risaralda, Caldas and Quindio.

The health system adopted in Colombia is the General Health Social Insurance (GHSI) based on managed competition and with two kinds of insurance: a private contributory scheme and the state-subsidised one for low-income people. Also, 92.4% of the Colombian population is covered by the GHSI, with a larger proportion in the subsidized system. Both contribution schemes have different health provider companies, with the public one having a lower coverage of health services and lower accessibility than the private one. The states of the Amazon region have fewer health provider companies compared with other states [15, 16].

### Study population and variables

This study population is comprised of all HIV/AIDS morbid cases and AIDS-related deaths, reported to the Public Health Surveillance System (PHSS) and to the Department of National Administration and Statistics (DNAS) from the Colombia National Institute of Health. Only information containing Colombian home addresses, occurring between 2008 and 2016, was included in this study.

We collected the following variables: age, sex, year of diagnosis and death, province and city of residence. Data were double-checked to remove inconsistencies and redundancies.

### Statistics analyses

#### Temporal trend analysis

We employed joinpoint regression to analyse changes in the annual incidence of HIV/AIDS, as well as in AIDS mortality rates, during the period of the study. All analyses were done in the Joinpoint Trend Analysis software 4.8.0.1 (National Cancer Institute, Calverton, MD. USA) and steps have been described previously [17]. Briefly, both incidence and mortality rates were directly age-adjusted following the joinpoint regression model. Joinpoint regression continues to add joinpoints in a linear trend until the number of joints cumulate to distinguish two different periods from one trend. The best-fitting adjusted model was assessed by the Monte Carlo permutation test. Here, we considered annual percent changes (APC), 95% confidence interval (CI 95%) and *p*-value. Upward and downward trends were considered only with positive or negative APC, respectively, and with *p*< 0.05. If these conditions were not met, it was considered a stationary trend.

All data were grouped by year, sex, counties, region and age range. The age ranges (years) were categorised into four groups: 0–14, 15–44, 45–64 and 65 and over. These categories were made on the basis of available information in the DNAS database for these age groups. The annual incidence of HIV/AIDS and AIDS mortality was calculated on the basis of annual population projections for the whole country and regions, and for the specific age groups and sexes. Both rates were standardised by 100,000 inhabitants.

We considered the incidence and mortality coefficients as the dependent variables. The study years were considered the independent variable.

#### Spatial analysis

We analysed the spatial distribution of HIV/AIDS incidence and AIDS mortality rates and their spatial autocorrelation and Kernel intensity estimator. To avoid the annual variation in the reported cases of HIV/AIDS and AIDS-related deaths, we grouped the data in 3-year periods: 2008–2010, 2011–2013 and 2014–2016.

Municipal HIV/AIDS incidence and AIDS mortality rates were analysed through choropleth maps. Both rates were calculated for each Colombian city on the basis of their average population projection for each 3-year period. The results were standardised for 100,000 inhabitants.

To assess the overall trend pattern of these variables, we analysed the spatial autocorrelation incidence and mortality rates using Global Moran’s I. The Global Moran’s l index ranges from − 1 to 1: an index of − 1 means dispersion, 0 is random behaviour and 1 means perfect association. However, to localize the clusters, we used the Local Indicator of Spatial Association (LISA) method. In LISA maps, we can identify four different spatial relationship groups of the analysed variable. High–high and low–low aggregations are areas with a high or low value of the variable under study surrounded by neighbouring areas, which have like values above or below average, respectively, of the specific variable. By contrast, high–low or low–high groups are areas with a high or low value of the analysed variable surrounded by neighbouring areas with opposite values below or above average of the same variable, respectively. We used the standardized first-order queen neighbours and the *p*-value obtained from 999 permutations as the definition of ‘neighbourhood’. We only considered spatial dependency with Global’s I index (I) above 0 and with *p*< 0.05 [18].

The spatial distribution and the autocorrelation analyses were conducted using the software ArcGis 10.6.1 (ESRI, Redland, CA. USA). The maps were constructed in the Universal Transverse Mercator Coordinate System (UTM), datum D_Bogota, on a scale of 1:12.000.000.

To analyse the direction of expansion or contraction of the HIV epidemic in Colombia, we used the Kernel density estimator. This method allows estimation of a density of an event occurring in one area that can influence its neighbourhood. Events occurring in places closer to each other receive a higher weight than events occurring in distant places [19]. Kernel analysis employs the adaptive influence radius and quartic Kernel smoothing function using the software TerraView 4.2.2 (INPE, Sao Paulo, SP. BR). We considered the municipalities as units of analysis.

## Results

From 2008 to 2016, Colombia recorded 72,994 cases of HIV/AIDS, and most of these cases were men (men: 53,432 cases, 73.20%; women: 19,561 cases, 26.80%). HIV was the preponderant diagnosis compared with AIDS in both men and women (men: 42,787 HIV, 10,645 AIDS; women: 16,322 HIV; 3240 AIDS). During the same period of the study, 21,515 AIDS-related deaths were recorded by the Health Ministry with a higher frequency in men than in women (men: 16,515 deaths, 76.76%; women: 5000 deaths, 23.24%). The Andean, Caribbean and Pacific Colombian regions had the highest recorded absolute frequency of HIV/AIDS cases (Table 1) and AIDS-related deaths (Table 2), of which the cases were mostly in the 15–44 age group. The main form of HIV transmission in the 0–14 age group was vertical transmission, while that in the other age groups was heterosexual transmission (Supporting Information, Table S1).

**Table 1:** Number of reported cases of HIV/AIDS for men and women in Colombia, 2008–2016

AGE (years old)	COLOMBIAN REGIONS					
	Amazon	Andean	Caribbean	Orinoquia	Pacific	Total of reported cases
**Men**						
0–14	6	155	217	15	75	468
15–44	476	25,156	8908	1087	6186	41,813
45–64	111	5446	2661	174	1645	10,037
65+	9	572	323	12	198	1114
**Women**						
0–14	20	164	219	15	75	493
15–44	316	6470	5262	492	2880	15,420
45–64	66	1609	1021	88	604	3388
65+	8	117	80	3	53	261

**Table 2:** Number of reported AIDS-related mortality in men and women in Colombia, 2008–2016

Age (years old)	COLOMBIAN REGIONS					
	Amazon (***n***=384)	Andean (***n***=10,827)	Caribbean (***n***=5521)	Orinoquia (***n***=853)	Pacific (***n***=3930)	Total of reported cases
**Men**						
0–14	1	30	49	5	14	99
15–44	181	5068	2538	402	1769	9958
45–64	58	3027	1306	176	976	5543
65+	11	483	224	26	171	915
**Women**						
0–14	3	34	45	4	12	98
15–44	89	1484	967	183	664	3387
45–64	36	627	337	53	292	1345
65+	5	74	55	4	32	170

### Temporal trend analyses

The annual HIV/AIDS incidence and AIDS mortality age-adjusted rates are shown in Tables 3 and 4, respectively. Both rates were higher for men and in the Caribbean region. Annual HIV/AIDS incidence and AIDS mortality age-adjusted rates were higher in the 15–44 and 45–64 age groups, respectively.

**Table 3:** Annual HIV/AIDS Incidence age-adjusted rates in Colombia, 2008–2016

VARIABLES	YEARS								
	2008	2009	2010	2011	2012	2013	2014	2015	2016
**Colombia**	12.35	14.13	14.96	15.91	16.35	17.4	19.62	21.92	23.14
**Sex**									
Men	17.25	19.53	21.11	23.28	23.85	25.67	29.51	34.12	26.51
Women	7.66	8.96	9.06	8. 83	9.12	9.42	10.02	10.03	9.64
**Age (years old)**									
0–14	0.78	1.24	1.02	0.96	0.73	0.84	0.64	0.6	0.6
15–44	21.09	23.64	24.7	26.21	27.53	29.13	33.72	37.54	40.1
45–64	12.15	14.4	15.85	17.09	16.35	17.53	17.72	20.3	20.34
65+	2.62	3.01	4.35	4.28	4.23	4.51	5.74	6.04	6.28
**Regions**									
Caribbean	13.15	17.39	17.63	18.38	21.22	24.23	25.15	27.32	26.72
Pacific	13.26	14.5	19.01	15.47	15.66	13.5	15.92	18.89	19.7
Andean	12	13.27	13.01	15.69	15.41	16.53	19.32	21.39	23.43
Orinoquia	9.4	10.29	13.41	9.92	9.87	14.01	14.75	18.74	20.77
Amazon	10.87	8.45	10.87	10.75	10.1	10.66	12.19	14.38	14.4

**Table 4:** Annual AIDS-mortality age-adjusted rates in Colombia, 2008–2016

VARIABLES	YEARS								
	2008	2009	2010	2011	2012	2013	2014	2015	2016
**Colombia**	5.44	5.2	5.35	5	5.15	5.13	5.08	5.17	5.5
**Sex**									
Men	8.56	8.11	8.24	7.97	7.65	8.01	7.8	7.9	8.53
Women	2.48	2.44	2.62	2.48	2.49	2.42	2.53	2.61	2.66
**Age (years old)**									
0–14	0.24	0.33	0.23	0.21	0.22	0.11	0.15	0.19	0.09
15–44	7.79	7.24	7.45	7.05	6.76	6.73	6.51	6.72	6.91
45–64	8.88	8.54	8.8	8.82	8.38	8.71	8.87	8.6	9.51
65+	2.97	3.25	3.23	2.63	3.07	4.36	4.22	4.35	5.18
**Regions**									
Caribbean	6.64	6.32	6.2	5.94	6.17	6.56	6.5	6.55	6.77
Pacific	6.16	5.81	6.14	6.1	5.9	5.65	5.67	6.1	6.59
Andean	4.8	4.62	4.7	4.61	4.21	4.43	4.35	4.31	4.63
Orinoquia	5.67	5.7	6.98	4.94	7.44	5.77	5.84	7.05	7.64
Amazon	4.66	4.44	5.51	3.99	3.37	4.87	5.14	4.4	4.26

Table 5 shows the temporal analysis of both rates. The incidence rate had an upward trend in the entire country and in all age groups except for the 0–14 age group (decreasing trend). The Caribbean, Andean, Orinoquia and Amazon regions also had an upward trend. Joinpoint regression identified two different periods in trend for the 15–44 age group with a higher APC in the second period than in the first (2008–2013: APC 6.2%, *p*=0.000; 2013–2016: APC 11.2%, *p*=0.000).

**Table 5:** Temporal trend analysis of HIV/AIDS incidence and AIDS mortality age-adjusted rates in Colombia, 2008–2016

		INCIDENCE		MORTALITY	
	Joinpoint results	APC (CI 95%)	P	APC (CI 95%)	P
**Colombia**		7.8 (6.7; 8.9)	0.000	−0.1 (−1.1; 1.0)	0.707
Men		9.8 (8.5; 11.1)	0.000	−0.3 (−1.5; 0.9)	0.464
Women		2.5 (1.1; 3.9)	0.005	0.7 (−0.3; 1.6)	0.158
**Age (years)**					
0–14		−7.4 (−12.4; −2.0)	0.014	−9.7 (−16.6; −2.3)	0.018
15–44		8.3 (7.1; 9.5)	0.000	−1.7 (−2.8; −0.6)	0.005
	2008–13	6.6 (4.6; 8.6)	0.000	−2.8 (−4.2; 1.2)	0.006
	2013–16	11.2 (7.5; 14.9)	0.000	3.2 (−5.9; 13.1)	0.410
45–64		5.5 (3.7; 7.3)	0.000	0.6 (−0.5; 1.7)	0.342
65+		10.2 (6.9; 13.5)	0.000	7.5 (3.6; 11.6)	0.002
**Regions**					
Caribbean		8.6 (6.3; 10.9)	0.000	0.6 (−0.6; 1.9)	0.317
	2008–11	–	–	− 2.9 (−6.3; 0.7)	0.079
	2011–16	–	–	2.4 (0.8; 4.0)	0.014
Pacific		3.2 (−0.8; 7.3)	0.113	0.4 (−1.2; 2.0)	0.661
Andean		8.8 (7.2; 10.4)	0.000	−0.9 (− 2.1; 0.3)	0.090
Orinoquia		10.2 (5.4; 15.3)	0.001	2.8 (−1.4; 7.2)	0.172
Amazon		5.1 (1.9; 8.5)	0.008	−0.6 (−5.0; 4.0)	0.736

Different from the incidence, the AIDS mortality rate was stabilised in Colombia in both sexes and in all regions. The mortality rate had a decreasing trend in the 0–14 and 15–44 groups. However, in the 15–44 group, the joinpoint regression distinguished two different periods with a decreasing trend in the first period and stabilisation in the second one (2008–2013: APC − 2.8%, *p*= 0.006; 2013–2016: 3.2%, *p*=0.41). The same trend also occurred in the Caribbean region, where two periods were identified with different behaviours: between 2008 and 2011 the mortality rate was stable (APC − 2.9%, *p*=0.079) and increased after 2011 (2011–2016: APC 2.4%, *p*=0.014).

The annual HIV/AIDS incidence and AIDS mortality age-adjusted rates for men and women are shown in Tables 6 and 7, respectively. Both rates were higher in 15–44 age group for men and women and in the Caribbean region.

**Table 6:** Annual HIV/AIDS incidence aged-adjusted rates for men and women in Colombia, 2008–2016

VARIABLES	YEARS								
	2008	2009	2010	2011	2012	2013	2014	2015	2016
**MEN**									
**Age (years old)**									
0–14	0.7	1.23	1.13	0.85	0.65	0.64	0.68	0.52	0.67
15–44	28.75	31.73	33.75	37.39	39.43	42.23	50.06	58.62	64.4
45–64	19.08	22.72	25.21	27.75	25.57	27.71	28.18	31.12	31.35
65+	5	5.23	7.6	7.67	7.92	8.73	9.67	10.92	11.39
**Region**									
Caribbean	16.83	21.48	21.68	23.89	27.87	31.02	32.78	36.96	37.56
Pacific	16.95	18.33	26.14	21.07	21.91	19.06	23.17	28.16	30.07
Andean	18.06	19.99	20.1	24.8	24.2	26.63	31.44	35.98	39.96
Orinoquia	11.14	12.81	16.62	13.28	12.74	18.4	20.79	27.76	31.04
Amazon	12.49	8.4	10.14	12.1	10.54	13.82	14.05	19.09	20.2
**WOMEN**									
#**Age (years old)**									
0–14	0.87	1.25	0.91	1.07	0.81	1.05	0.6	0.68	0.52
15–44	13.61	15.7	15.81	15.18	15.75	16.13	17.45	16.5	15.78
45–64	5.8	6.79	7.32	7.41	7.98	8.32	8.26	10.54	10.41
65+	0.64	1.17	1.67	1.5	1.23	1.08	2.54	2.1	2.17
**Regions**									
Caribbean	9.56	13.39	13.69	13	14.73	17.61	17.68	17.82	16.03
Pacific	9.78	10.88	12.22	10.14	9.69	8.16	8.96	9.86	9.52
Andean	6.25	6.88	6.26	7	6.96	6.83	7.59	7.23	7.31
Orinoquia	7.63	7.55	10.17	6.55	6.99	9.62	8.71	9.71	10.49
Amazon	9.19	8.5	11.59	9.34	9.65	7.44	10.29	9.59	8.52

**Table 7:** Annual AIDS-mortality age-adjusted rates in Colombia for men and women in Colombia, 2008–2016

VARIABLES	YEARS								
	2008	2009	2010	2011	2012	2013	2014	2015	2016
**MEN**									
**Age (years old)**									
0–14	0.15	0.19	0.18	0.26	0.23	0.17	0.12	0.15	0.05
15–44	11.8	11.03	10.87	10.32	9.65	9.75	9.59	9.73	10.04
45–64	15.3	14.37	15.07	14.84	14.51	14.83	14.22	14.05	15.9
65+	5.62	5.46	5.93	5.14	5.32	8.47	7.69	8	9.53
**Regions**									
Caribbean	10.21	9.83	9.52	8.91	9.07	10.08	9.81	9.86	10.11
Pacific	8.76	8.33	8.9	8.74	7.97	7.73	7.56	8.37	9.12
Andean	8.02	7.56	7.51	7.53	6.95	7.37	7.09	6.99	7.73
Orinoquia	8.66	7.74	9.66	7.04	11.01	7.73	9.25	9.81	10.99
Amazon	5.37	5.11	7.42	5.09	4.44	7.43	5.98	5.89	5.5
**WOMEN**									
**Age (years old)**									
0–14	0.33	0.47	0.28	0.16	0.22	0.05	0.17	0.24	0.14
15–44	3.88	3.52	4.1	3.82	3.9	3.73	3.44	3.71	3.78
45–64	3.01	3.21	3.08	3.34	2.82	3.16	4.04	3.68	3.76
65+	0.77	1.42	1.01	0.58	1.23	1.02	1.4	1.4	1.69
**Regions**									
Caribbean	3.16	2.91	2.97	3.04	3.33	3.14	3.28	3.34	3.55
Pacific	3.74	3.45	3.55	3.63	3.98	3.74	3.9	4.02	4.27
Andean	1.79	1.86	2.09	1.88	1.66	1.7	1.8	1.81	1.74
Orinoquia	2.64	3.62	4.29	2.84	3.87	3.81	2.45	4.3	4.33
Amazon	3.93	3.75	3.54	2.84	2.28	2.25	4.29	2.9	2.99

The temporal analysis of annual HIV/AIDS incidence and AIDS mortality age-adjusted rates for men and women are shown in Table 8. An increasing trend in HIV/AIDS incidence in men in all Colombian regions and age groups above 15 years was observed. A decreasing trend was observed only in the 0–14 age group (APC − 7.5%, *p*=0.040). The joinpoint regression detected two periods of time in the 15–44 age group with high APC in the second period (2008–2013: APC 8.2%, *p*=0.000; 2013–2016: APC 15.0%, *p*=0.000). Similarly, the incidence rate among women had a downward trend in the 0–14 age group, but a sole upward trend was detected in the 45 years and above age group (45–64 years: APC 7.0%, *p*=0.000; 65 years and over: APC 11.0%, *p*=0.024). The incidence among women increased only in the Caribbean and Andean regions (APC 5.9%, *p*=0.006; APC 1.9%, *p*=0.014, respectively), as opposed to that among men.

**Table 8:** Temporal trend analyses of annual HIV/AIDS incidence and AIDS mortality age-adjusted rates for men and women in Colombia, 2008–2016

		INCIDENCE		MORTALITY	
	Joinpoint results	APC (CI 95%)	P	APC (CI 95%)	P
**MEN**					
**Age (years old)**					
0 a 14		−7.5 (−14.0; −0.4)	0.040	−6.0 (−16.5; 5.8)	0.261
15 a 44		10.8 (9.2; 12.5)	0.000	−2.2 (−3.5; −1.0)	0.003
	2008–12	–	–	−4.8 (−6.6; −2.9)	0.002
	2008–13	8.2 (6.2; 10.3)	0.000	–	–
	2012–16	–	–	0.5 (− 1.5; 2.5)	0.650
	2013–16	15.0 (11.4; 18.8)	0.000	–	–
45 a 64		5.0 (3.1; 7.1)	0.000	0.0 (− 1.3; 1.4)	0.869
65+		10.1 (7.6; 12.7)	0.000	7.8 (3.6; 12.1)	0.003
**Regions**					
Caribbean		10.1 (8.4; 11.8)	0.000	0.3 (− 1.2; 1.8)	0.821
Pacific		5.5 (1.1; 10.1)	0.023	− 0.3 (− 2.4; 1.7)	0.600
Andean		10.6 (8.9; 12.4)	0.000	− 0.8 (−2.2; 0.5)	0.149
Orinoquia		13.7 (8.6; 19.0)	0.000	2.9 (−1.5; 7.4)	0.186
Amazon		9.2 (4.0; 14.6)	0.003	0.5 (−5.1; 6.5)	0.867
**WOMEN**					
**Age (years old)**					
0–14		−7.0 (−13.0; −0.6)	0.035	−11.2 (−20.9; − 0.4)	0.045
15–44		1.6 (0.0; 3.3)	0.069	−0.5 (−2.2; 1.1)	0.404
45–64		7.0 (5.0; 9.0)	0.000	3.2 (0.4; 6.0)	0.033
65+		11.1 (1.9; 21.1)	0.024	6.6 (−1.0; 14.9)	0.081
**Regions**					
Caribbean		5.9 (2.3; 9.6)	0.006	1.9 (0.6; 3.2)	0.010
Pacific		−2.1 (−5.2; 1.1)	0.139	2.1 (0.8; 3.3)	0.007
Andean		1.9 (0.6; 3.2)	0.014	−1.0 (−3.0; 1.0)	0.250
Orinoquia		3.5 (−1.1; 8.3)	0.125	2.9 (− 3.3; 9.5)	0.316
Amazon		−0.8 (−4.8; 3.3)	0.613	−2.4 (−8.8; 4.3)	0.400

The only change detected in AIDS mortality rate in men was in the age group 65 years and above with a significantly increasing trend (APC 7.8%, *p*=0.003). However, although the general trend in the 15–44 age group was stable, the joinpoint regression identified two different periods with a decreasing trend in the first period (2008–2012: APC − 4.8%, *p*=0.002) and a stabilization in the second one (2012–2016: APC 0.5%, *p*=0.65). Among women, a decreasing trend in the 0–14 age group (APC − 11.2%, *p*=0.045) and increasing trend in the 45–64 age group (APC 3.2%, p-0.033) in the Caribbean and Pacific regions was detected (APC 1.9%, *p*=0.010; APC 2.1%, *p*=0.007, respectively).

### Spatial analysis

**Fig. 2:**
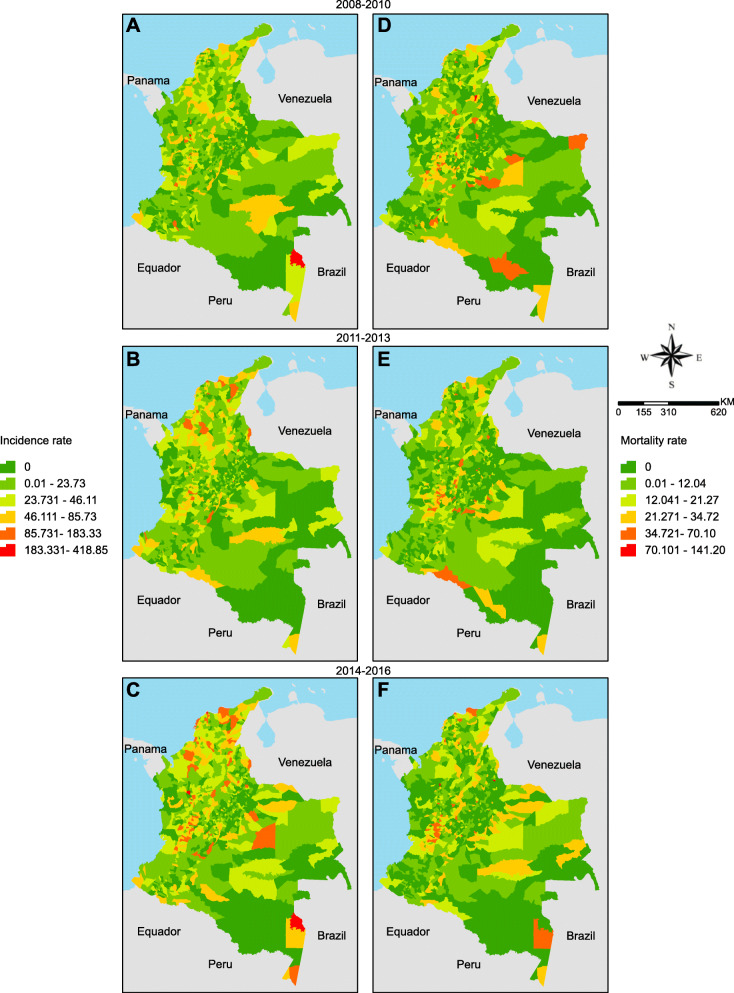
Spatial distribution of HIV/AIDS incidence (**a**, **c**, **e**), AIDS mortality rates (**b**, **d**, **e**). The maps were constructed using ArcGis version 10.6.1

Figure 2 shows the spatial distribution of HIV/AIDS incidence (Fig. 2a, b and c) and AIDS mortality rates (Fig. 2d, e and f). HIV/AIDS incidence showed a territorial expansion, while AIDS mortality showed contraction. The most impacted municipalities were in the Caribbean, Andean and Orinoquia regions. After 2014, the incidence and mortality rates increased in municipalities bordering Venezuela in the Caribbean and Orinoquia regions.

**Fig. 3:**
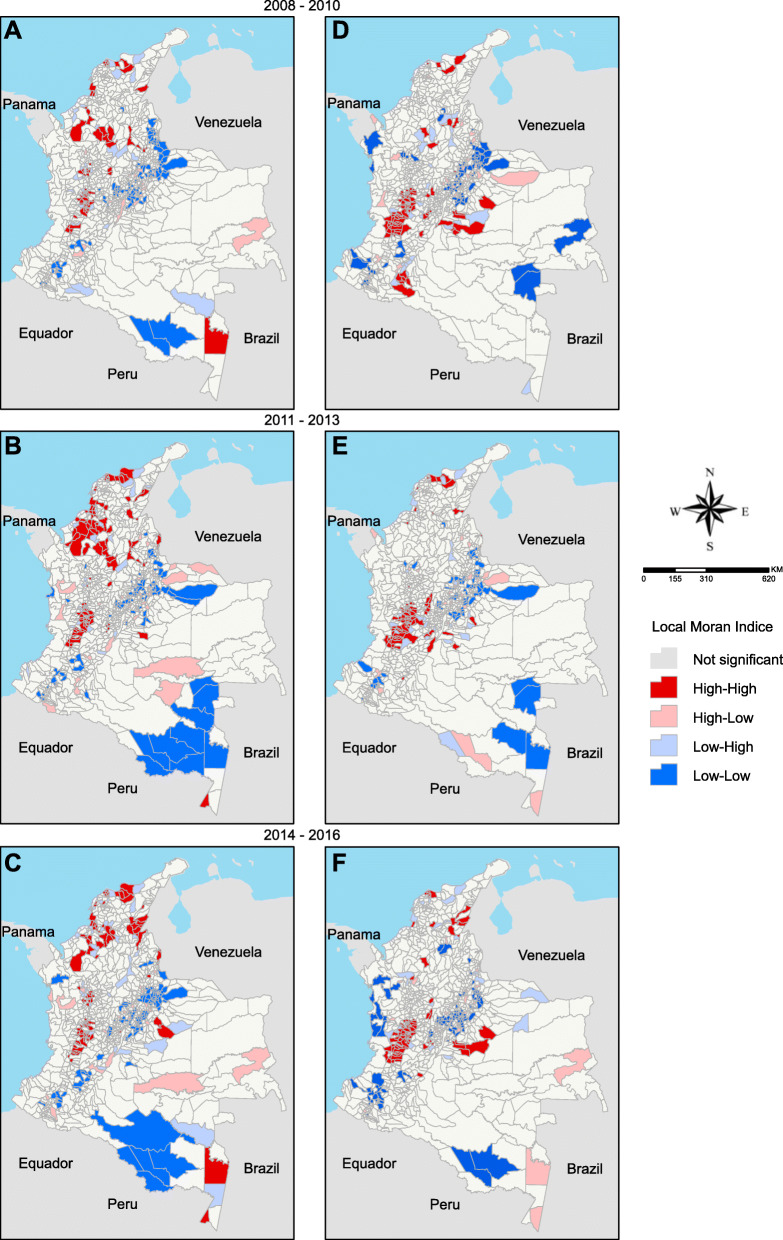
LISA maps of HIV/AIDS incidence (**a**, **c**, **e**) and AIDS mortality rates (**b**, **d**, **e**). The maps were constructed using ArcGis version 10.6.1

All univariate spatial autocorrelations of Global Moran’s I indexes were positive and statistically significant in terms of both HIV/AIDS incidence and AIDS mortality (HIV/AIDS incidence: 2008–2010 - I=0.15 *p*=0.00, 2011–2013 - I=0.28 *p*=0.00; 2014–2016 - I=0.22 *p*=0.00; AIDS mortality: 2008–2010 - I=0.26 *p*=0.00; 2011–2013 - I=0.24 *p*=0.00; 2014–2016 - I=0.23 *p*=0.00). Figure 3a, b and c show the LISA maps for the HIV/AIDS incidence and Fig. 3d, e and f for AIDS mortality rates. The high–high clusters for both rates were located on the Caribbean coast and in the ‘coffee belt’ of the Andean region. The low–low clusters were restricted to the Orinoquia and Amazon regions. Also, between 2014 and 2016, a high–high cluster of incidence and mortality appeared in the Caribbean and Orinoquia regions bordering Venezuela (Fig. 3c and f).

**Fig. 4:**
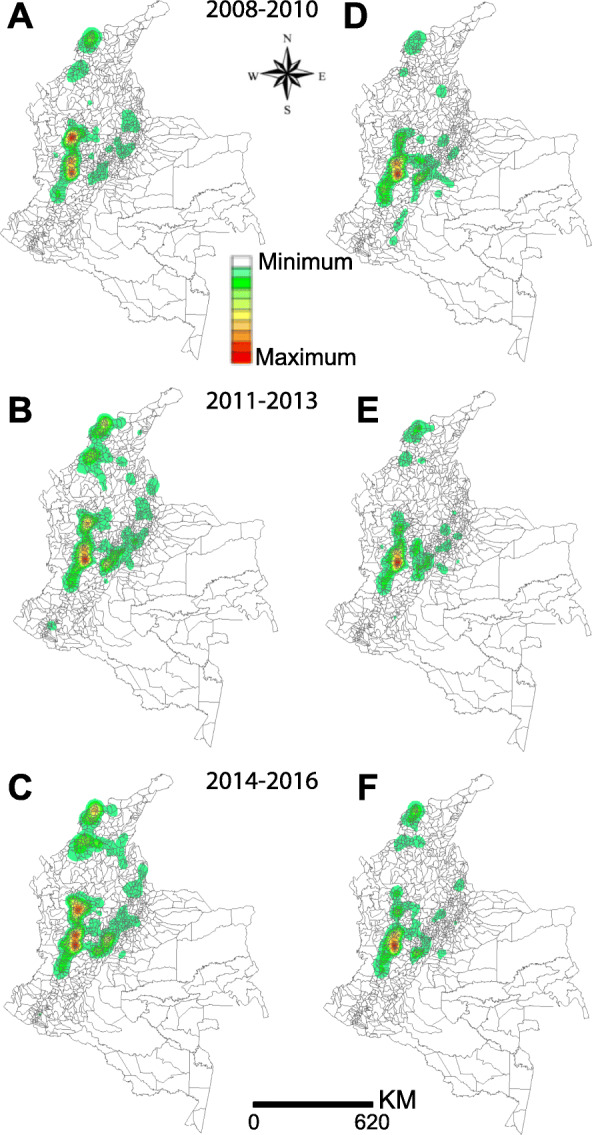
Kernel maps of HIV/AIDS incidence (**a**, **c**, **e**) and AIDS mortality rates (**b**, **d**, **f**). The maps were constructed using ArcGis version 10.6.1 and TerraView version 4.2.2

Figure 4 shows the Kernel maps of HIV/AIDS incidence (Fig. 4a, b and c) and AIDS mortality rates (Fig. 4d, e and f). The density was higher in municipalities of the ‘coffee belt’ than in others for both incidence and mortality rates. HIV/AIDS incidence expanded from the north of the Caribbean to the northeast and midwest of Colombia, while AIDS mortality contracted in zones towards the Andean region’s ‘coffee belt’.

## Discussion

This study showed a temporal and spatial panoramic view of the HIV epidemic in Colombia. Although HIV/AIDS incidence showed an upward trend in the period of the study, the AIDS mortality rate was stable. The impact of the HIV epidemic was more remarkable in men than in women in the 15–44 and 65 years and above age groups. Among men, the epidemic had an increasing trend in all Colombian regions; among women, this trend was only in the Andean and Caribbean regions.

The HIV epidemic in Colombia follows the observation from all LA with increased HIV/AIDS incidence by 7% from 2010 to 2018 [2]. However, this phenomenon is not only particular to LA: between 2017 and 2018, Canada also reported an increase of 8.2% in new HIV infections [20]. These figures suggest the necessity of further investing in public policy in HIV control [6]. By contrast, South Africa had the largest investment in ART and reports a substantial decrease in new HIV infections [21].

Further action is necessary in Colombia to reach the 2030 UNAIDS goals through the 90–90–90 targets. Even after a health system restructuring in 2008 that guaranteed more access to ART [8], Colombia still ranks low on various indices among all LA countries in terms of overall ARV coverage, HIV testing and PLWHA population proportion with suppressed viral load. In addition, by the end of 2018, Colombia still had not adopted the Pre-Exposure Prophylaxis (PrEP) to HIV [6, 22]. Countries that adopted the Universal Treatment and Test for HIV policy (UTT) have observed an increasing number of PLWHA with suppressed viral load and decreasing HIV incidence and AIDS-related deaths [23, 24]. The fact that our AIDS mortality data shows a downward trend only from 2008 to 2013 among men in the 15–44 age group, followed by a stabilization, should be a cause of concern among health authorities.

We observed an increasing trend in HIV/AIDS incidence occurring earlier in men than in women and the inverse occurring in AIDS mortality. Gender inequities are obstacles in the fight against HIV in women worldwide. Societal, cultural, economic and legal inequities increase the vulnerability to HIV/AIDS among women. These factors can explain the late diagnosis for HIV and earlier mortality among women than among men [25, 26]. Other like examples include: In 2018, 49,929 women were diagnosed with HIV in the Union European World Health Organization region, and more than 50% of them were diagnosed late [27]. In South Africa, ART coverage was lower among women who were in abusive relationships [28]. Furthermore, in the United States, women suffering from poverty stigma had lower ART adherence and lower frequency of HIV care visits than in others [29].

The high incidence of HIV/AIDS and AIDS mortality in the Caribbean and Andean regions are attributed to the status of these regions as main tourist destinations in Colombia. In the Dominican Republic, where tourism is the main contributor to the gross domestic product, the prevalence of HIV is approximately 1% [30]. Colombia is one of the countries in the world with a high rate of sexual tourism associated with gender and social inequities that exacerbates the HIV epidemic. Most of Colombian PLWHA are poor, have low educational attainment and have minimal access to health care and secure jobs. Social inequities have led people to work in the sexual tourism industry to sustain themselves and their families [31]. The HIV prevalence among sex workers in Colombia is also moderately high at 1.2% due not only to societal stigma but also to political and structural factors [32]. Preventing HIV in sex workers is essential to decrease HIV overall in Colombia. In West Africa, approximately 75% of HIV infection in heterosexual men are correlated with sexual intercourse with female sex workers [33].

The decreasing trends observed in HIV/AIDS incidence and AIDS mortality in the 0–14 age group reflects a downwards mother-to-child HIV transmission. In 2003, the Colombian Health Ministry launched the project ‘Elimination Strategy of mother-to-child HIV and Syphilis transmission’. This strategy guarantees antenatal care, with multiple tests for HIV and syphilis, including treatment for pregnant women, their partners and babies, caesarean delivery and implementation of formula milk breastfeeding. From 2008 to 2016, mother-to-child HIV transmission decreased from 5.8 to 2.6% [34]. A recent study showed a high percentage of pregnant women with HIV who started antenatal care late, as well as late ARV treatment and viral load exam after the 34th week of pregnancy [35]. Cuba invested in the same strategy, and it was the first country in the world to eliminate mother-to-child HIV transmission, followed by Thailand, Armenia and Belarus [36]. To reach the OMS goals [37] further effort is needed in Colombia.

The increasing trend in HIV/AIDS incidence among the 65 years and above age group should be a cause of concern among health authorities. Although this trend may be due to late HIV diagnosis, it may also be attributed to unprotected intercourse due to low level of knowledge about HIV/STI transmission and underestimated risk for infection. A study in South Korea showed that sexually active elderly people, mainly men, had multiple sexual partners and a very low rate of condom use. Furthermore, the elderly who had a high knowledge of STI prevention and transmission had lower risk behaviour [38]. In the US, between 2008 and 2016, 757 people aged 65 years and above were diagnosed with HIV, and 92.6% of them reported having not used a condom. In addition, 15.9% of them related at least one risk behaviour for HIV infection, with exchanging sex for money as the most prevalent one [39].

This work is an ecological study. Therefore, we cannot claim causality between HIV transmission, demographic groups, migration, stigma or other social phenomena. Various confounders may be overlooked in these types of analyses. In addition, this study was limited by particular missing data due to the sources of information. However, this represents a small proportion (576 cases of HIV/AIDS and 312 deaths) of the entire database that did not compromise the analyses. Finally, this work was also limited by the employment of two particular datasets (PHSS and DNAS) without identifiers, thereby making it impossible to extend analyses between variables.

## Conclusions

Our study found an upward trend in HIV/AIDS incidence and a stable trend in the AIDS mortality rate in Colombia. The decreasing trend in HIV/AIDS incidence and AIDS mortality rate in the 0–14 age reflects downwards mother-to-child HIV transmission. The upward trend in HIV/AIDS incidence and AIDS mortality rates in older and younger women, respectively, compared with those in men, may be due to late diagnosis and treatment. The Caribbean and the ‘coffee belt’ regions were the most impacted by the HIV epidemic which may be due to the sexual tourism.

Our study provides information to Colombian health authorities in order to fight HIV transmission. This effort is much more than just testing and making ART or PrEP universally available. It is also necessary to combat social discrimination against HIV, to promote gender equality, to guarantee human rights to sex workers as well as discriminalization of prostitution, and to promote universal access to healthcare services. Lastly, our study showed the necessity and the urgency of establishing STI prevention programs for elderly people.

## Supplementary Information

**Additional file 1: Table S1.** Number of reported cases of HIV/AIDS by the main possible forms of HIV transmission, 2008-2016.

## Data Availability

The cartographic bases used in this study are publically available on the website of the Institute of Geography Augustin Codazzi (https://www.igac.gov.co). Population databases were taken from DANE website (http://www.dane.com.co). The maps in Figs. 1, 2, 3, 4 were constructed by the authors of this paper. The data that support the findings regarding the compulsory notification of HIV/AIDS are available from the Colombia National Institute of Health and the data about mortality from the National Statistic Administrative Department. Restrictions apply to the availability of these data, which were used under license for the current study, and so are not publicly available.

## Abbreviations

AIDS: Acquired immunodeficiency syndrome
APC: Annual percentual change
ART: Antiretroviral Therapy
DNAS: Department of National Administration and Statistics
HIV: Human Immunodeficiency Virus
LA: Latin America
LISA: Local indicator of spatial autocorrelation
PLWHA: People living with HIV and AIDS
PHSS: Public Health Surveillance System
PrEP: Pre-exposure prophylaxis
STI: Sexually transmitted infections

## References

[1] UNAIDS (2019). Global HIV & AIDS statistics — 2019 fact sheet.

[2] GBD 2017 HIV collaborators (2019). Global, regional, and national incidence, prevalence, and mortality of HIV, 1980–2017, and forecasts to 2030, for 195 countries and territories: a systematic analysis for the Global Burden of Diseases, Injuries, and Risk Factors Study 2017. Lancet HIV.

[3] Trickey A, May TM, Vehreschild JJ, Obel N, Gill J, Crane HM (2017). Survival of HIV-positive patients starting antiretroviral therapy between 1996 and 2013: a collaborative analysis of cohort studies. Lancet HIV.

[4] Piñeirúa A, Sierra-Madero J, Cahn P, Guevara Palmero RN, Martínez Buitrago E, Young B (2015). The HIV care continuum in Latin America: challenges and opportunities. Lacet Infec Dis.

[5] Crabtree-Ramírez B, Belaunzarán-Zamudio PF, Cortes CP, Morales M, Sued O, Sierra-Madero J (2020). The HIV epidemic in Latin America: a time to reflect on the history of success and the challenges ahead. J Int AIDS Soc.

[6] Djellouli N, Quevedo-Gómes MC (2015). Challenges to successfull implementation of HIV and AIDS related health policies in Cartagena, Colômbia. Soc Sci Med.

[7] Rivillas JC, Devia Rodriguez R, Song G, Martel A (2018). How do we reach the girls and women who are the hardest to reach? Inequitable opportunities in reproductive and maternal health care services in armed conflict and forced displacement settings in Colombia. PLoS One.

[8] Arrieta-Gómez AI (2018). Realizing the fundamental right to health through litigation: the Colombian case. Health Hum Rights.

[9] Addressing the HIV crisis among Venezuelan migrants in Colombia (2019). Center for Global Health.

[10] Rodríguez-Morales AJ, Bonilla-Aldana DK, Morales M, Suárez JA, Martínez-Buitrago E (2019). Migration crisis in Venezuela and its impact on HIV in other countries: the case of Colombia. Ann Clin Microbiol Antimicrob.

[11] Velásquez J, Contreras L, Contreras C, Martinez N, Chaparro J, Sarmiento C (2016). Prevalência de infección por VIH en Bogotá, D.C., Colombia, en 2012. Caracterización por localicdades. Rev Fac Med.

[12] Dwyer-Lindgren L, Cork MA, Sligar A, Steuben KM, Wilson KF, Provost NR (2019). Mapping HIV prevalence in sub-Saharan Africa between 2000 and 2017. Nature..

[13] Rebolledo EAS, Chiaravalloti F, Giatti LL (2018). Experiences, benefits and challenges of the use of geoprocessing for the devellopment of primary health care. Rev Panam Salud Publica.

[14] Statista (2020). Income distribution inequality based on Gini coefficient in Latin America as of 2017, by country.

[15] Garcia-Subirats I, Vargas I, Mogollón-Pérez AS, De Paepe P, Silva MRF, Unger JP (2014). Inequities in access to health care in different health systems: a study in municipalities of Central Colombia and North-Easter Brazil. Int J Equity Health.

[16] Indicadores Básicos de Colombia. 2015. Ministerio de Salud. https://www.minsalud.gov.co/sites/rid/Lists/BibliotecaDigital/ride/vs/ed/gcfi/indicadores-basicos-en-salud-2015.pdf. Accessed 01 Oct 2020.

[17] Kim HJ, Fay MP, Feuer EJ, Midthune DN (2000). Permutation tests for joinpoint regression with applications to cancer rates. Stat Med.

[18] Quin Q, Wei G, Tang W, Mahapatra T, Wang L, Zhang N (2017). Spatial analysis of the human immunodeficiency virus epidemic among men who have sex with men in China, 2006-2015. Clin Infect Dis.

[19] Sousa AIA, Pinto Junior VL (2016). Spatial and temporal analysis of AIDS cases in Brazil, 1996-2011: increased risk areas over time. Epidemiol Serv Saude.

[20] Haddad N, Robert A, Weeks A, Popovic N, Siu W, Archibald C (2019). Hiv in Canadá – surveillance report, 2018. Can Commun Dis Rep.

[21] Vandormael A, Cuadros D, Kim HY, Bärnighaussen T, Tanser F (2020). The state of the HIV epidemic in rural KwaZulu-Ntal, South Africa: a novel application of disease metrics to assess trajectories and highlight areas intervention. Int J Epidemiol.

[22] Luz PM, Veloso VG, Grinsztejn B (2019). The HIV epidemic in Latin America: accomplishments and challenges on treatment and prevention. Curr Opin HIV AIDS.

[23] Havlir D, Lockman S, Ayles H, Larmarange J, Chamie G, Gaolathe T (2020). What do the universal test and treat trials tell us about the path to HIV epidemic control?. J Int AIDS Soc.

[24] Wyk VPV, Msemburi W, Dorrington RE, Laubscher R, Groenewald P, Bradshaw D (2019). HIV/AIDS mortality trends pre and post ART for 1997–2012 in South Africa – have we turned the tide?. S Afr Med J.

[25] Rosin C, Elzi L, Thurnheer C, Fehr J, Cavassini M, Calmy A (2015). Gender inequalities in the response to combination antirretroviral therapy over time: the Swiss HIV cohort study. HIV Med.

[26] Girum T, Wasie A, Lentiro K, Muktar E, Shumbei T, Difer M (2018). Gender disparity in epidemiological trend of HIV/AIDS infection and treatment in Ethiopia. Achives Public Health.

[27] Mardh O, Quinten C, Kuchukhidze G, Seguy N, Dara M, Amato-Gauci AJ (2019). HIV among women in the WHO European region – epidemiological trends and predictor of late diagnosis, 2009-2018. Euro Suveill.

[28] Pulerwitz J, Gottert A, Kahn K, Haberland N, Julien A, Selin A (2019). Gender norms and HIV testing/treatment uptake: evidence from a large population-based sample in South Africa. AIDS Behav.

[29] Leddy AM, Turan JM, Johnson MO, Neilands TB, Kempf MC, Konkle-Parker D (2019). Poverty stigma is associated with suboptimal HIV care and treatment outcomes among women living with HIV in the U.S. AIDS..

[30] Burgos JFC, Padilla M, Nuñez A, Varas-Días N, Matiz-Reyes A (2019). An ethnographic study of ‘touristic escapism’ and health vulnerability among Dominican male tourism workers. Glob Public Health.

[31] Quevedo-Gómez MC, Krumeich A, Abadía-Barrero CE, Borne HW (2019). Social inequalities, sexual tourism and HIV in Cartagena, Colombia: an ethnographic study. BMC Public Health.

[32] UNAIDS (2020). AIDSinfo: Trend of new HIV infection.

[33] Alary M, Lowndes CM (2004). The central role of clients of female sex workers in the dynamics of heterosexual HIV transmission in sub-Saharan Africa. AIDS..

[34] Colômbia (2018). Boletim Epidemiológico de Salud.

[35] Arango-Ferreira C, Villegas DI, Burbano LD, Quevedo A (2019). Follow up of HIV perinatal exposure and accomplishment of strategies to reduce the risk of viral transmission, experience in a reference hospital in Medellín. Biomedica..

[36] WHO. Towards a HIV-free generation in Cuba. Bull World Health Organ. 2016;94(12):866–7. 10.2471/BLT.16.021216 PMC5153925 27994278

[37] Taylor M, Newman L, Ishikawa N, Laverty M, Hayashi C, Ghidinelli M (2017). Elimination of mother-to-child transmission of HIV and syphilis (EMTCT): Processs, progress and program integration. PLoS Med.

[38] Kim HY, Choe HS, Lee DS, Yoo JM, Lee SJ (2019). Sexual behavior and sexually transmitted infection in the elderly population of South Korea. Investig Clin Urol.

[39] Okara E, Mason S, Xia M (2018). Too old to test? Prevalence and correlates of HIV testing among sexually active older adults. J Gerontol Soc Work.

