# Correction to: The HIV epidemic in Colombia: spatial and temporal trends analysis

**DOI:** 10.1186/s12889-021-10800-1

**Published:** 2021-04-20

**Authors:** Jhon Freddy Montana, Glenda Roberta Oliveira Naiff Ferreira, Carlos Leonardo Figueiredo Cunha, Ana Angélica Rêgo de Queiroz, Wellington Augusto Andrade Fernandes, Sandra Helena Isse Polaro, Lucia Hisako Takase Gonçalves, Danielle Costa Carrara Couto, Elucir Gir, Renata Karina Reis, Wiliam Sorensen, Eliã Pinheiro Botelho

**Affiliations:** 1 Nursing Graduate Program, Federal University of Para, Rua Augusto Correia, 01, Complexo Saúde, Guamá, Belém, Para 66075-110 Brazil; 2 Nursing Department of Federal University of Rio Grande do Norte, Centro das Ciências da Saúde, Campus Universitário Lagoa Nova, Natal, Rio Grande do Norte 59078-970 Brazil; 3 Laboratory of Spatial Analyzes (LAENA), Center for Amazonina Studies (NAEA), Federal University of Para, Rua Ausgusto Correia, 01, Complexo Engenharia, Guamá, Belém, Para 66075-110 Brazil; 4 School of Technology in Geoprocessing, Federal University of Pará, Rua Augusto Correia, 01, Complexo Engenharia, Guamá, Belém, Para 66075-110 Brazil; 5 Graduate Program of Fundamental Nursing. Nursing School of Ribeirao Preto, University of Sao Paulo, Av dos Bandeirantes, 3900. Campus Universitario – Monte Alegre, Ribeirao Preto, Sao Paulo 14040-902 Brazil; 6 Department of Health & Kinesiology, University of Texas at Tyler, 3900 University Blvd., Tyler, TX 75799 USA

**Correction to: BMC Public Health 21, 178 (2021)**

**https://doi.org/10.1186/s12889-021-10196-y**

It was highlighted that the original article [1] contained an error in the third paragraph of the Background section. This Correction article shows the incorrect and correct sentence. The original article has been updated.

**Incorrect**

The Colombian Caribbean region has the second-highest prevalence of HIV/AIDS in the world after sub-Saharan Africa. By the end of 2019, there were 330,000 Colombian PLWHA, 6,900 AIDS-related deaths and 13,000 new HIV infections recorded in the same year.

**Correct**

Colombia is part of the Caribbean region, which has the second-highest prevalence of HIV/AIDS in the world after sub-Saharan Africa. In this region, by the end of 2019, there were 330,000 PLWHA, 6,900 AIDS-related deaths and 13,000 new HIV infections recorded in the same year.
