# Supplementary material for: The HIV epidemic in Colombia: spatial and temporal trends analysis
Source: BMC Public Health. 2021 Jan 21;21:178. doi: 10.1186/s12889-021-10196-y (PMC7818909; doi:10.1186/s12889-021-10196-y)
Supplement: Supplementary file 1 — Additional file 1: Table S1. Number of reported cases of HIV/AIDS by the main possible forms of HIV transmission, 2008-2016. [file 12889_2021_10196_MOESM1_ESM.docx]

Table S1 – Number of reported cases of HIV/AIDS by the main possible forms of HIV transmission, 2008-2016.

| **ROUTES OF HIV TRANSMISSION** | **AGE (YEARS)** | | | |
| --- | --- | --- | --- | --- |
|  | **0-14**  **(n=961)** | **15-44**  **(n=57.233)** | **45-64**  **(n=13,425)** | **65+**  **(n=1,375)** |
| Occupational accident | 2 | 54 | 14 | 0 |
| Men who have sex with men | 45 | 20,118 | 2,357 | 129 |
| Heterosexual | 193 | 34,694 | 10,456 | 1,201 |
| Vertical transmission | 606 | 79 | 14 | 0 |
| Organ transplants | 7 | 28 | 13 | 2 |
| Blood transfusions | 12 | 24 | 4 | 3 |
| Injection Drug Use | 2 | 150 | 32 | 1 |
| Ignored | 94 | 2,086 | 535 | 39 |
